# Abnormal cerebrospinal fluid composition can accompany central nervous system involvement in COVID-19

**DOI:** 10.1055/s-0042-1758378

**Published:** 2022-11-09

**Authors:** Josef Finsterer, Fulvio Alexandre Scorza

**Affiliations:** 1 Neurology & Neurophysiology Center, Vienna, Austria.; 2 Universidade Federal de São Paulo, Escola Paulista de Medicina, Disciplina de Neurociência, São Paulo SP, Brazil.

Dear Editor,

We read with interest the review article by Domingues about the cerebrospinal fluid (CSF) findings among coronavirus disease 2019 (COVID-19) patients with central nervous system (CNS) involvement. 1 The 663 patients included from 75 studies comprised patients with stroke (n = 25), encephalitis (n = 81), encephalopathy (n = 264), headache (n = 52), inflammatory syndrome (n = 56), meningitis (n = 4), and seizures (n = 22). 1 It was concluded that CSF analysis has a role in evaluating COVID-19-associated CNS disorders and may contribute to a better understanding of the relationship between severe acute respiratory syndrome coronavirus 2 (SARS-CoV-2) infection and the CNS. 1 The study is attractive but raises concerns that should be discussed.

We disagree that the categories stroke, encephalitis, encephalopathy, meningitis, seizures, and headache can be called “syndromes” 1 . Mixing diseases with symptoms and symptoms with syndromes is confusing. Because seizures and headache are symptoms, the underlying CNS cause should be identified.

It should be explained why certain CNS conditions, such as subarachnoid bleeding (SAB), venous sinus thrombosis (VST), cerebral vasculitis, posterior reversible encephalopathy syndrome (PRES), cerebellitis, hypophysitis, opsoclonus myoclonus ataxia syndrome, reversible cerebral vasoconstriction syndrome (RCVS), multiple sclerosis (MS), transverse myelitis, and delirium were not included in the review. Because CSF findings in these CNS pathologies may be specific, a lot of information might have been missed. At least some of the patients with these disorders have undergone a spinal tap. 2

It is not comprehensible why the 6 patients with encephalopathy and a polymerase chain reaction (PCR) positive for SARS-CoV-2 were classified as encephalopathy and not as encephalitis. Encephalitis does not necessarily go along with a structural CNS lesion. 3 Even the CSF leukocyte count can be normal in these patients. 3 Therefore, the definition of encephalopathy (CNS manifestations of systemic disease in the absence of abnormalities on cerebral imaging) is misleading.

Twenty-two patients with seizures were included. 1 We should know the underlying neurologic disease and the indication for a spinal tap in patients with seizures. Was encephalitis/meningitis suspected in these patients?

Surprisingly, patients with multisystem inflammatory syndrome in children (MIS-C) were excluded from the evaluation, although there was no age-limit. 1 However, patients with inflammatory syndrome (n = 56) were included. This discrepancy should be solved. Furthermore, a definition of inflammatory syndrome (n = 56) should be provided. Did this group only comprise patients with acute disseminated encephalomyelitis (ADEM), neuromyelitis optica (NMO), cerebral isolated syndrome (CIS), and acute, hemorrhagic leucoencephalitis (AHLE), or were other types of inflammatory syndrome included as well?

There is a discrepancy between the total number of patients included (n = 663) and the sum of patients assigned to the 7 categories (n = 514). What about the missing 149 patients? Which category of syndrome were they assigned to?

It is not comprehensible why Guillain-Barre syndrome (GBS) was excluded. 1 A subtype of GBS can go along with brainstem encephalitis (Bickerstaff encephalitis). 4

Overall, the interesting study has some limitations and inconsistencies that call the results and their interpretation into question. Addressing these limitations could further strengthen and reinforce the statement of the study. Exclusion and inclusion criteria should be reproducible, and the entire spectrum of CNS involvement in COVID-19 should be evaluated.
